# Supplementary material for: Multicenter study of bleeding and thromboembolic events with durvalumab tremelimumab vs. atezolizumab and bevacizumab in advanced HCC
Source: JHEP Rep. 2026 Mar 11;8(6):101818. doi: 10.1016/j.jhepr.2026.101818 (PMC13141955; doi:10.1016/j.jhepr.2026.101818)
Supplement: Multimedia component 1 [file mmc1.pdf]

Multicenter study of bleeding and thromboembolic events with durvalumab ± tremelimumab *vs.* atezolizumab and bevacizumab in advanced HCC

Najib Ben Khaled, Raphael Mohr, Leonie Jochheim, Valentina Zarka, Monika Karin, Fabian Artusa, Julia M. Schütte, Vera Himmelsbach, Ursula Ehmer, Katrin Böttcher, Friedrich Foerster, Simon Johannes Gairing, Paula Bark, Alexander Weich, Ignazio Piseddu, Monika Rau, Bernhard Scheiner, Lorenz Balcar, Marino Venerito, Philipp Heumann, Arne Kandulski, Catherine Leyh, Christoph Roderburg, Tom Lüdde, Matthias Pinter, Julia Mayerle, Jens U. Marquardt, Fabian Finkelmeier, Enrico N. De Toni, Andreas Geier, Florian P. Reiter

Table of contents

Table S1…………………………………………………………………………………...2

Fig. S1……………………………………………………………………………………...4

Table S1. Bleeding type. Bleeding type of A/B versus DT/D (n=325 vs. n=165). This table annotates the number of patients experiencing a specific bleeding event. For descriptive analyses of bleeding type, all bleeding events were included, with the possibility of patients contributing more than one event (for example a patient experiencing both epistaxis and esophageal variceal bleeding, both events are included).

**Bleeding types of A/B versus DT/D**			
**Bleeding name**	**A/B n=325 patients**	**DT/D n=165 patients**	**Overall n=490 patients**
Epistaxis, n (%)	17 (5.2)	2 (1.2)	19 (3.9)
Esophageal variceal bleeding, n (%)	10 (3.1)	4 (2.4)	14 (2.9)
Lower GI bleeding, n (%)	9 (2.8)	1 (0.6)	10 (2)
GI bleeding unspecified, n (%)	6 (1.8)	3 (1.8)	9 (1.8)
Upper GI bleeding unspecified, n (%)	6 (1.8)	1 (0.6)	7 (1.4)
Bleeding unspecified, n (%)	4 (1.2)	2 (1.2)	6 (1.2)
Gingival bleeding, n (%)	6 (1.8)	0 (0)	6 (1.2)
Gastric ulcer bleeding, n (%)	3 (0.9)	2 (1.2)	5 (1)
Gastric bleeding, n (%)	1 (0.3)	3 (1.8)	4 (0.8)
Upper GI bleeding with reflux esophagitis, n (%)	2 (0.6)	2 (1.2)	4 (0.8)
Duodenal ulcer bleeding, n (%)	3 (0.9)	0 (0)	3 (0.6)
Intracerebral bleeding, n (%)	3 (0.9)	0 (0)	3 (0.6)
Gastric variceal bleeding, n (%)	2 (0.6)	0 (0)	2 (0.4)
Hematuria, n (%)	2 (0.6)	0 (0)	2 (0.4)
Hemorrhoidal bleeding, n (%)	1 (0.3)	1 (0.6)	2 (0.4)
Suspected lower GI bleeding, n (%)	2 (0.6)	0 (0)	2 (0.4)
Bleeding from gastric tumor infiltration, n (%)	1 (0.3)	0 (0)	1 (0.2)

Liver bleeding, n (%)	1 (0.3)	0 (0)	1 (0.2)
Midgut bleeding, n (%)	1 (0.3)	0 (0)	1 (0.2)
Peripheral bleeding after vascular surgery, n (%)	1 (0.3)	0 (0)	1 (0.2)
Rectal varices bleeding, n (%)	0 (0)	1 (0.6)	1 (0.2)
Retroperitoneal hematoma, n (%)	1 (0.3)	0 (0)	1 (0.2)
Suspected upper GI bleeding, n (%)	1 (0.3)	0 (0)	1 (0.2)
Tumor and duodenal bleeding, n (%)	1 (0.3)	0 (0)	1 (0.2)
Tumor bleeding, n (%)	1 (0.3)	0 (0)	1 (0.2)
Vaginal bleeding, n (%)	1 (0.3)	0 (0)	1 (0.2)

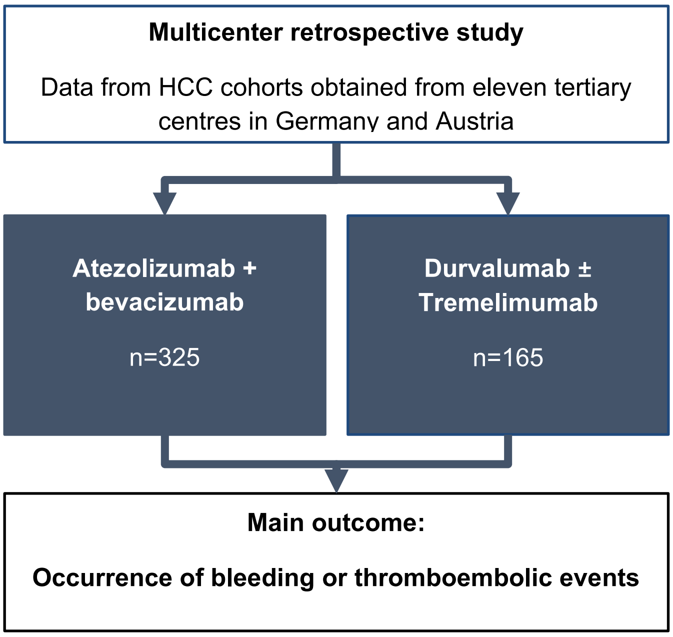

Fig. S1. Study flowchart.
