# Supplementary material for: Multicenter study of bleeding and thromboembolic events with durvalumab tremelimumab vs. atezolizumab and bevacizumab in advanced HCC
Source: JHEP Rep. 2026 Mar 11;8(6):101818. doi: 10.1016/j.jhepr.2026.101818 (PMC13141955; doi:10.1016/j.jhepr.2026.101818)
Supplement: Multimedia component 4 [file mmc4.pdf]

Multicenter study of bleeding and thromboembolic events with durvalumab ± tremelimumab *vs.* atezolizumab and bevacizumab in advanced HCC

Authors

Najib Ben Khaled, Raphael Mohr, Leonie S. Jochheim, …, Enrico N. De Toni, Andreas Geier, Florian P. Reiter

Correspondence

Reiter_F@ukw.de (F.P. Reiter).

Graphical abstract

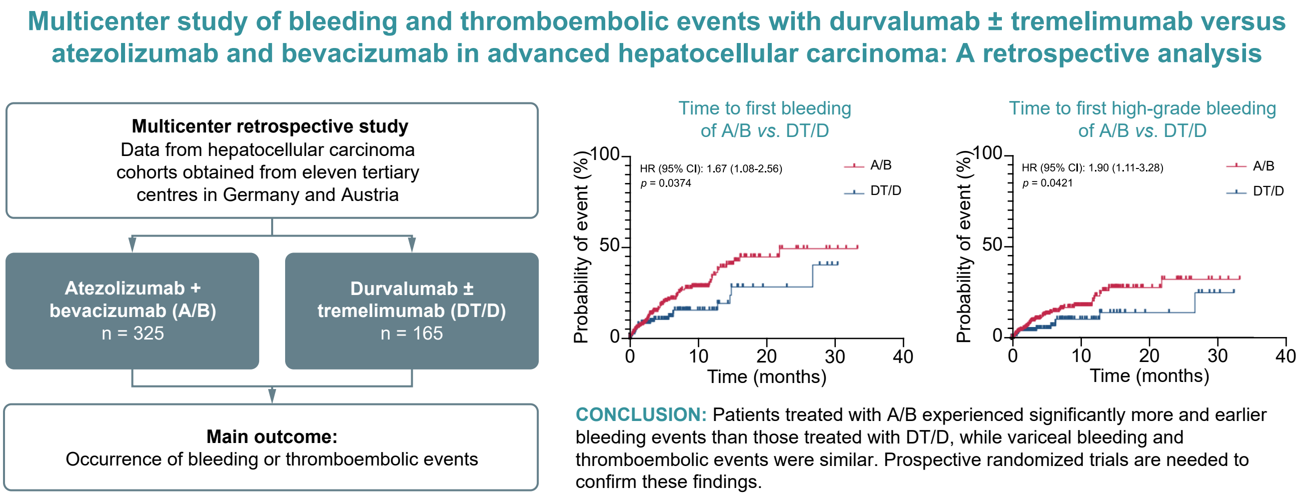

Highlights:

- Atezolizumab/bevacizumab was associated with a higher incidence of overall and high-grade bleeding events compared with durvalumab±tremelimumab in advanced HCC.
- Bleeding events occurred earlier in patients treated with atezolizumab/bevacizumab.
- Treatment with atezolizumab/bevacizumab was independently associated with increased bleeding risk in multivariate analysis.
- Rates of variceal bleeding and thromboembolic events did not differ significantly between treatment groups.
- Findings should be interpreted cautiously because of the retrospective design and baseline imbalances.

Impact and implications:

This multicenter retrospective study was scientifically justified by the need to clarify whether the addition of VEGF inhibition to PD-L1–based immunotherapy confers differential bleeding or thromboembolic risks in patients with advanced HCC. The results are particularly relevant for clinicians and trialists, as they demonstrate a higher incidence and earlier onset of overall and high-grade bleeding events with atezolizumab/bevacizumab compared with durvalumab ± tremelimumab, despite similar rates of variceal bleeding and thromboembolic events. These findings may inform treatment selection, risk stratification, and surveillance strategies in routine practice, especially in patients with portal hypertension or other bleeding risk factors. Given the retrospective design and baseline-imbalances between treatment groups, the results should be interpreted cautiously and warrant confirmation in prospective randomized studies or, if not feasible, through meta-analyses.

https://doi.org/10.1016/j.jhepr.2026.101818
© 2026 The Authors. Published by Elsevier B.V. on behalf of European Association for the Study of the Liver (EASL). This is an open access article under the CC BY license (http://creativecommons.org/licenses/by/4.0/).

Multicenter study of bleeding and thromboembolic events with durvalumab ± tremelimumab *vs*. atezolizumab and bevacizumab in advanced HCC

Najib Ben Khaled[1], Raphael Mohr[2], Leonie S. Jochheim[3,4], Valentina Zarka[5], Monika Karin[1], Fabian Artusa[2], Julia M. Schütte[3], Vera Himmelsbach[6], Ursula Ehmer[7], Katrin Böttcher[7,8,9], Friedrich Foerster[10], Simon Johannes Gairing[10], Paula Bark[10], Alexander Weich[11], Ignazio Piseddu[1], Monika Rau[5], Bernhard Scheiner[12], Lorenz Balcar[12], Marino Venerito[13], Philipp Heumann[14], Arne Kandulski[14], Catherine Leyh[15], Christoph Roderburg[15], Tom Lüdde[15], Matthias Pinter[12], Julia Mayerle[1], Jens U. Marquardt[16], Fabian Finkelmeier[6], Enrico N. De Toni[1], Andreas Geier[5], Florian P. Reiter[5,*]

JHEP Reports **2026**. vol. 8 | 1–9

Check for updates

Background & Aims: Atezolizumab/bevacizumab (A/B) and durvalumab ± tremelimumab (DT/D) are preferred first-line regimens for the treatment of hepatocellular carcinoma (HCC). Although both therapies share an anti-PD-L1-backbone, only the A/B-combination is targeting vascular-endothelial-growth-factor (VEGF). This difference may have safety-implications, as the anti-VEGF component may elevate the risk of bleeding and thromboembolic events. This multicenter study analyzes bleeding or thromboembolic events in patients receiving therapy with DT/D, compared with A/B.

Methods: Patients with HCC treated with A/B exclusively before the approval of DT/D, or treated with DT/D, were recruited from 11 tertiary centers and analyzed retrospectively. Information on baseline characteristics was collected. Kaplan–Meier analyses and multivariate Cox regression modeling was used to compare event rates.

Results: This study included 490 patients (n = 165 DT/D; n = 325 A/B). The DT/D group showed a higher prevalence of gastroesophageal varices and increased spleen size. Median follow-up time was similar in both groups. A total of 74 patients (22.8%) treated with A/B showed a bleeding event of any grade *vs*. 22 patients (13.3%) treated with DT/D ($p = 0.016$). High-grade ≥3 bleeding episodes were more frequent in the A/B group with 47 patients (14.5%) *vs*. 12 patients (7.3%) in the DT/D group ($p = 0.027$). Kaplan–Meier analysis showed significantly shorter time to first bleeding ($p = 0.037$) with A/B as compared with DT/D. Multivariate regression confirmed that treatment with A/B was independently associated with an increased bleeding risk ($p = 0.010$). Variceal bleeding and thromboembolic events did not differ significantly between both groups.

Conclusions: Overall and high-grade bleeding events were more frequent and occurred earlier in patients treated with A/B *vs*. DT/D, whereas variceal bleeding, grade 4–5 bleeding, and thromboembolic events did not differ significantly between groups. These findings should be confirmed in randomized trials or, if not feasible, through meta-analyses to provide more robust evidence for treatment decisions in advanced HCC.

© 2026 The Authors. Published by Elsevier B.V. on behalf of European Association for the Study of the Liver (EASL). This is an open access article under the CC BY license (http://creativecommons.org/licenses/by/4.0/).

Introduction

Liver cancer is a leading cause of cancer-related death worldwide.[1,2] Hepatocellular carcinoma (HCC) accounts for >75% of all primary liver cancers, with an increasing incidence.[3,4] Despite recommended surveillance strategies by several guidelines,[4,5] most cases of HCC are diagnosed at or progress to an advanced stage,[6,7] where systemic therapy is indicated.[8]

Immune checkpoint inhibitor (ICI)-based regimens were transformative for the treatment of patients with unresectable HCC and became the first-line standard by demonstrating superiority over tyrosine kinase inhibitor (TKI) monotherapy for advanced HCC.[9–12] As of July 2025, three ICI-based combination therapies are approved in the USA and Europe: atezolizumab plus bevacizumab (A/B),[9,13] durvalumab with or without tremelimumab (DT/D),[10,14,15] and, most recently, ipilimumab plus nivolumab,[11] which received approval shortly before the time of writing.

The combination of A/B demonstrated superiority in terms of progression-free survival (PFS) and median overall survival (mOS) over sorafenib in the pivotal IMbrave150 trial, leading to its approval in 2020.[9] Mechanistically, atezolizumab targets programmed death-ligand 1 (PD-L1), whereas bevacizumab inhibits vascular-endothelial-growth-factor (VEGF).[9] The

* Corresponding author. Address: Division of Hepatology, Department of Medicine II, University Hospital Würzburg, Oberdürrbacher Straβe 6, 97080 Würzburg, Germany. Tel.: +49 931 2010; fax: +49 931 201 640023.
E-mail address: Reiter_F@ukw.de (F.P. Reiter).
‡ Partner site - Bavarian Cancer Research Center (BZKF).
† Partner site - German Alliance for Liver Cancer (GALC).
https://doi.org/10.1016/j.jhepr.2026.101818

second immunotherapeutic option, consisting of DT/D, gained approval by the EMA in 2023 based on the results of the HIMALAYA trial.[10] DT/D significantly improved mOS as compared to sorafenib.[10] Monotherapy with durvalumab was also approved, based on its non-inferiority compared with sorafenib, whereas superiority was demonstrated only for the combination of durvalumab and tremelimumab.[10] An important mechanistic difference between the two regimens is that the DT/D combination constitutes a purely ICI-based therapy, targeting PD-L1 and cytotoxic T-lymphocyte-associated protein 4 (CTLA-4).[10] The addition of the anti-VEGF agent bevacizumab to standard therapies has been associated with increased bleeding events and thromboembolic complications across various malignancies.[16,17] This raises concerns about its safety in patients with HCC,[18–20] who commonly present with underlying chronic liver disease or cirrhosis, potentially increasing the susceptibility to bleeding[21] and thromboembolic events.[22]

Our group has recently published data comparing A/B with the TKI lenvatinib in the treatment of HCC, focusing on bleeding and thromboembolic events.[23] In this analysis, we did not observe significant differences in the incidence of these complications.[23] This finding might be attributed to the potent anti-VEGF activity of the multikinase inhibitor lenvatinib, potentially leading to a similar incidence of vascular events as compared to bevacizumab-containing regimen.[24]

To investigate whether anti-VEGF free therapies carry a lower risk of bleeding or thromboembolic side effects, we performed an analysis of DT/D in a large, multicenter, European real-world cohort.

The primary objective of this study was to examine the characteristics of patients receiving DT/D in comparison to A/B, and to evaluate the occurrence of bleeding and thromboembolic events associated with the two standard ICI-based therapies, placing special emphasis on variceal bleeding, the most severe bleeding complication in the context of chronic liver disease.

Patients and methods

Patient population

The study included patients with unresectable HCC treated with DT/D or A/B from 10 German and one Austrian tertiary care center. The data from the A/B cohort come from a historical cohort[23] that was recruited until March 2023, before the approval of D/T in Europe. The patients in the A/B group started therapy between June 2019 and March 2023, whereas the DT/D group started between April 2023 and April 2025. The data cut-off was performed in September 2025 for the DT/D group. The diagnosis of HCC was based on histopathological findings or typical diagnostic imaging, following the criteria outlined by EASL.[25,26] The study was approved by the local authorities (Ethics Committee at Julius-Maximilians-University Würzburg, 156/21-me) and followed the principles of the Declaration of Helsinki. We used the STROBE cohort checklist and adhered to the ESMO Guidelines for Reporting Oncology Real-World Evidence.[27,28] Data were analyzed retrospectively.

Treatments

Patients received the following treatment regimens: A/B, involving intravenous administration of atezolizumab at a dosage of 1,200 mg and bevacizumab at 15 mg per kg of body weight every 3 weeks.[9] DT/D was either administered according to the STRIDE protocol with a single dose of tremelimumab at 300 mg and repeated durvalumab at 1,500 mg every 4 weeks,[10] or in case of durvalumab monotherapy at 1,500 mg durvalumab every 4 weeks.[10] In cases of intolerance, doses of the specified therapy protocols could be delayed at the investigators' discretion. Throughout the treatment, patients were consistently monitored using clinical, laboratory, and imaging assessments, in accordance with the standard of care and following the current HCC guidelines.[29] Follow-up was conducted for all patients.

Study parameters

A comprehensive analysis of predisposing factors, such as variceal status, spleen size, platelet count, and anticoagulation, was performed. To investigate the factors influencing the selection of DT/D, we analyzed the reasons for treatment choice by reviewing the medical records.

Study design, data source, and study data management

In this multicenter retrospective study data were extracted from medical records and patient reports during the recruitment phase for observational, prospective patient cohorts at each participating center. Each center provided anonymized source data via a predefined form. The analysis identified no duplicated cases, likely because of the diverse geographical locations. The dataset compilation occurred in September 2025, after which data completeness was verified, followed by comprehensive quality control and validation procedures.

Exclusion criteria

Therapy decisions were made at the discretion of the physician. To ensure a genuine real-world context, no exclusions were made among HCC patients. Patients with combined HCC/cholangiocarcinoma (CCA) tumors or fibrolamellar HCC were not included in the study.

Statistical analysis and illustration

Statistical analyses were performed using GraphPad Prism version 10 (GraphPad Software, San Diego, CA, USA) and R studio software (RStudio Inc., Boston, MA, USA). Patient characteristics were summarized using descriptive statistics. The normality of continuous variables was assessed using the Shapiro–Wilk test. Continuous data were expressed as mean ± SD or median plus range, and compared using the *t* test or Mann–Whitney *U* test, as appropriate. Categorical variables were described as frequencies and proportions and compared using Fisher's exact test. Bleeding and thromboembolic event rates were analyzed as available retrospectively by the Common Terminology Criteria for Adverse Events (CTCAE) grade. For the analysis of bleeding toxicity, each patient was counted once and assigned the highest CTCAE grade observed. Time-to-event analyses were conducted for first bleeding and for first high-grade bleeding (CTCAE ≥3) using the Kaplan–Meier method, with comparisons via the log-rank test. For time-to-bleeding or time-to-thromboembolism, patients were censored at death, last follow-up, or stop of A/B or DT/D therapy. For descriptive analyses of bleeding type (*e.g.* epistaxis, intracranial bleeding, etc.), all bleeding events were

included, with the possibility of patients contributing more than one event. Median follow-up time was estimated with the reverse Kaplan–Meier method. Univariate and multivariate Cox proportional hazards models were used to estimate hazard ratios (HR) with 95% CIs for bleeding risk. Variables with significant association in univariate analysis were considered for multivariate models. Multicollinearity was assessed using variance inflation factor and R^2 with other variables. Variables with strong collinearity were excluded to ensure model stability. A *p* value <0.05 was considered statistically significant.

Results

Baseline characteristics

A total of 490 patients with HCC were included in the analysis set; 325 were treated with A/B and 165 with DT/D (Fig. S1). Median follow-up time was 11.0 months in the DT/D group *vs.* 14.0 in the A/B group (*p* = 0.11). Demographics were similar between both groups (Table 1). There were significantly more patients with non-viral etiology in the A/B group (A/B: *n* = 222 [68%], DT/D: n = 97 [59%], *p* = 0.045). Underlying liver cirrhosis was present in most patients (A/B: n = 233/325 [72%], DT/D: n = 132/165 [80%], *p* = 0.049), with a higher frequency of patients with Child–Pugh score B in the DT/D group (A/B: n = 40/233 [17%], DT/D: n = 36/132 [27%], *p* = 0.031). Availability of pre-treatment gastroscopy was similar in both groups, with no gastroscopy reported in 22 patients treated with A/B (7%) *vs.* seven patients with DT/D (4%, *p* = 0.315). Compared with the A/B cohort, there were more patients with varices overall (56% *vs.* 41%, *p* = 0.002) and with high-grade varices in the DT/D group (esophageal varices grade III A/B: 2%, DT/D: 18%, *p* <0.001). History of variceal bleeding was low in both groups (A/B: 6%, DT/D: 9%, *p* = 0.191). Mean spleen size was slightly higher in the DT/D group (A/B: 12.7 cm, DT/D: 13.7 cm, *p* = 0.002), whereas the platelet count was similar (*p* = 0.294). Use of anticoagulation or antiplatelets did not differ in both groups (anticoagulation in A/B: 29%, DT/D: 29%, *p* >0.999; antiplatelets in A/B: 26%, DT/D: 30%, *p* = 0.283). The majority of patients received the regimen as first-line therapy (A/B: 96%, DT/D: 92%, *p* = 0.066). Presence of macrovascular invasion was similar (A/B: 35%, DT/D: 34%, *p* = 0.915), whereas extrahepatic spread was more frequent in patients receiving A/B (A/B: 44%, DT/D: 32%, *p* = 0.017).

Frequency and severity of bleeding events

In total, 74 bleeding events were reported among the 325 patients treated with A/B (22.8%), compared with 22 events in

Table 1. Baseline characteristics.

Patient characteristics	A/B n = 325	DT/D n = 165	*p* value
Age, median (range)	69 (25–96)	69 (30–89)	0.5365
Sex, female, n (%)	75 (23)	29 (18)	0.1983
Cirrhosis, n (%)	233 (72)	132 (80)	**0.0489**
Child–Pugh A	175 (75)	87 (66)	0.0696
Child–Pugh B	40 (17)	36 (27)	**0.0313**
Child–Pugh C	13 (6)	6 (5)	0.8083
Unknown	5 (2)	3 (2)	>0.9999
Gastroesophageal varices, n (%)	134 (41)	93 (56)	**0.0016**
Esophageal grade I	85 (63)	46 (49)	**0.0410**
Esophageal grade II	40 (30)	30 (32)	0.7704
Esophageal grade III	3 (2)	17 (18)	**<0.0001**
Gastric or fundic	4 (3)	0 (0)	0.1463
Others (rectal or downhill varices)	2 (1)	0 (0)	0.5142
Therapy of varices, n (%)	109 (34)	66 (40)	0.1639
Non-selective β-blockers	67 (61)	30 (45)	**0.0428**
Banding	21 (19)	10 (15)	0.5452
Non-selective β-blockers + banding	21 (19)	26 (39)	**0.0048**
No EGD available, n (%)	22 (7)	7 (4)	0.3149
History of variceal hemorrhage, n (%)	19 (6)	15 (9)	0.1914
Spleen size, mean ± SD	12.7 ± 2.8	13.7 ± 2.5	**0.0019**
Platelets, mean ± SD	194.3 ± 115.1	179.1 ± 105.6	0.2939
Anticoagulation, n (%)	95 (29)	48 (29)	>0.9999
Antiplatelet agents, n (%)	83 (26)	50 (30)	0.2830
BCLC stage, n (%)			
BCLC A	6 (2)	4 (2)	0.7389
BCLC B	82 (25)	52 (32)	0.1632
BCLC C	224 (69)	102 (62)	0.1289
BCLC D	13 (4)	4 (2)	0.4432
Unknown	0 (0)	3 (2)	**0.0377**
First-line systemic therapy, n (%)	311 (96)	151 (92)	0.0662
Etiology of underlying liver disease, n (%)			
HBV/HCV	86 (26)	51 (31)	0.3379
Non-viral	222 (68)	97 (59)	**0.0447**
Unknown	17 (5)	17 (10)	0.0581
Extrahepatic spread, n (%)*	103 (44)	52 (32)	**0.0165**
Macrovascular invasion, n (%)*	83 (35)	55 (34)	0.9146

Continuous variables were compared using the Student's t-test or the Mann-Whitney *U* test, as appropriate, after assessment of normality distribution using Shapiro-Wilk test. Categorical variables were compared using Fisher's exact test. A/B, atezolizumab and bevacizumab; BCLC, Barcelona Clinic Liver Cancer; DT/D, durvalumab with or without tremelimumab; EGD, esophagogastroduodenoscopy; n, number.

*Data regarding extrahepatic spread and macrovascular invasion were available for 236 patients treated with A/B, data regarding macrovascular invasion for 160 patients treated with DT/D. Values in bold denote statistical significance.

the 165 patients treated with DT/D (13.3%) (Table 2), resulting in a significantly higher bleeding frequency in the A/B group (p = 0.016, odds ratio [OR] 1.92, 95% CI 1.14–3.22). When analyzing bleeding severity by CTCAE grade, grade 1 events occurred in 11 (3.4%) *vs.* 2 (1.2%) patients, and grade 2 events in 16 (4.9%) *vs.* 8 (4.8%) patients for A/B and DT/D, respectively. Grade 3 bleeding was more frequent in the A/B group (30 patients, 9.2%) compared with DT/D (five patients, 3.0%). Grade 4 and 5 bleeding were observed in eight (2.5%) and nine (2.8%) A/B patients, and in six (3.6%) and one (0.6%) DT/D patients, respectively. Overall, high-grade bleeding (CTCAE ≥3) occurred more frequently in the A/B group (47 patients, 14.5%) compared with DT/D (12 patients, 7.3%, p = 0.027, OR 2.16, 95% CI 1.11–4.19). In contrast, low-grade (grade 1–2) bleeding did not differ significantly between the two groups (p = 0.470, OR 1.40, 95% CI 0.66–2.98).

Anti-VEGF treatment seemed to be associated with specific bleeding events, particularly mucosal bleeding including epistaxis (5.2% of patients treated with A/B *vs.* 1.2% with DT/D), gingival bleeding (1.8% *vs.* 0%), vaginal bleeding (0.3% *vs.* 0%), and lower GI bleeding (2.8% *vs.* 0.6%). Bleedings related to portal hypertension, such as esophageal varices bleeding (3.1% with A/B *vs.* 2.4% with DT/D) and other GI bleeding events seemed to occur independently of VEGF inhibition in both therapies. In particular, the incidence of variceal hemorrhage did not differ significantly between the groups (p = 0.782).

Time to first bleeding was significantly shorter among patients treated with A/B compared with those receiving DT/D (log-rank p = 0.037) (Fig. 1A). This was also observed for time to first high-grade bleeding (CTCAE ≥3), which was significantly shorter in the A/B group *vs.* DT/D (log-rank p = 0.042) (Fig. 1B).

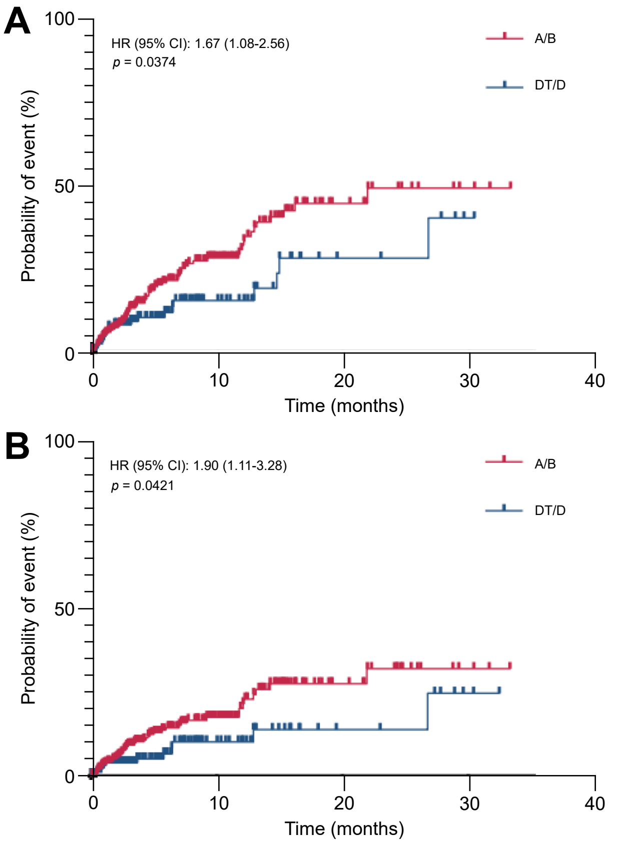

Fig. 1. Kaplan–Meier analysis of time-to-bleeding in both cohorts. Time to first bleeding episode (A), and time to first high-grade bleeding episode are displayed (B). The line represents the cumulative incidence. A/B, atezolizumab/bevacizumab; DT/D, durvalumab + tremelimumab/durvalumab; HR, hazard ratio (log-rank).

Risk factors of bleeding episodes

In the univariate Cox regression analysis, A/B treatment was associated with an increased risk of bleeding (HR 1.67, 95% CI: 1.04–2.78, p = 0.040) (Table 3). Surrogates of liver function were other significant univariate predictors of bleeding, such as presence of liver cirrhosis (HR 2.39, 95% CI: 1.38–4.50, p = 0.004) and Child–Pugh class C (HR 3.37, 95% CI: 1.16–7.76, p = 0.011). Markers of portal hypertension were also associated with an increased risk of bleeding in univariate analysis, including platelet count (HR 0.9977, 95% CI: 0.9953–0.9999, p = 0.047), spleen size (HR 1.12, 95% CI: 1.03–1.20, p = 0.005), history of variceal bleeding (HR 3.64, 95% CI: 1.96–6.26, p <0.001), and presence of high-grade varices grade 2–3 (HR 2.87, 95% CI: 1.74–4.71, p <0.001). Anticoagulation was also linked to increased risk of bleeding in univariate analysis (HR 1.81, 95% CI: 1.18–2.73, p = 0.006). Multivariate Cox regression analysis confirmed treatment with A/B as an independent predictor of bleeding episodes (HR 2.02, 95% CI: 1.21–3.55, p = 0.010) (Table 3). Prior variceal bleeding (HR 2.60, 95% CI: 1.28–4.94, p = 0.005), and presence of high-grade varices grade 2–3 continued to represent independent risk factors (HR 1.84, 95% CI: 1.02–3.30, p = 0.041). Unknown variceal status was also associated with a significantly increased bleeding risk (HR 3.13, 95% CI: 1.15–7.20, p = 0.013), underlining the importance of systematic endoscopic screening before treatment initiation.

Table 2. Frequency and severity of bleeding events of A/B *vs.* DT/D (n = 325 *vs.* n = 165).

CTCAE grade	A/B n = 325	DT/D n = 165	*p* value	Odds ratio	95% CI
Any bleeding, n (%)	**74 (22.8)**	**22 (13.3)**	**0.0157**	**1.92**	**1.14–3.22**
Grade 1–2 (non-severe), n (%)	27 (8.3)	10 (6.1)	0.4701	1.40	0.66–2.98
Grade ≥3 (severe), n (%)	**47 (14.5)**	**12 (7.3)**	**0.0268**	**2.16**	**1.11–4.19**
Grade 1, n (%)	11 (3.4)	2 (1.2)			
Grade 2, n (%)	16 (4.9)	8 (4.8)			
Grade 3, n (%)	30 (9.2)	5 (3.0)			
Grade 4, n (%)	8 (2.5)	6 (3.6)			
Grade 5, n (%)	9 (2.8)	1 (0.6)			

Comparisons between groups were performed using the Fisher's exact test. A/B, atezolizumab and bevacizumab; DT/D, durvalumab with or without tremelimumab. Values in bold denote statistical significance.

Table 3. Cox proportional hazards regression for risk of bleeding.

Variable	Univariate HR	95% CI	*p* value	Multivariate HR	95% CI	*p* value
A/B (DT/D as reference)	**1.67**	**1.04–2.78**	**0.0396**	**2.02**	**1.21–3.55**	**0.0101**
Age	0.99	0.97–1.01	0.4417			
Sex, male (female as reference)	1.15	0.7–2.01	0.601			
Cirrhosis (yes *vs.* no)	**2.39**	**1.38–4.5**	**0.0036**			
Child–Pugh class						
Child–Pugh B (A as reference)	1.52	0.82–2.65	0.1575	1.44	0.77–2.54	0.2344
Child–Pugh C (A as reference)	**3.37**	**1.16–7.76**	**0.0105**	2.55	0.84–6.22	0.0613
Platelets	**0.9977**	**0.9953–0.9999**	**0.0467**			
Spleen size	**1.12**	**1.03–1.2**	**0.0052**			
BCLC stage						
BCLC B (BCLC A as reference)	0.43	0.16–1.45	0.1156			
BCLC C (BCLC A as reference)	0.45	0.18–1.48	0.1212			
BCLC D (BCLC A as reference)	2.07	0.54–8.46	0.2811			
EHS (yes *vs.* no)	0.75	0.46–1.19	0.2312			
MVI (yes *vs.* no)	1.39	0.85–2.21	0.1764			
ECOG PS						
ECOG 1 (0 as reference)	0.8	0.5–1.25	0.3374			
ECOG 2 (0 as reference)	1.74	0.83–3.31	0.1141			
ECOG 3 (0 as reference)	2.3	0.13–10.65	0.4129			
First-line therapy (not first-line as reference)	1.62	0.67–5.29	0.3483			
Prior therapy (yes *vs.* no)	0.71	0.42–1.15	0.1802			
History of variceal bleeding (yes *vs.* no)	**3.64**	**1.96–6.26**	**<0.0001**	**2.6**	**1.28–4.94**	**0.0054**
Varices grade						
Varices grade 1 (no varices as reference)	1.47	0.86–2.46	0.1478	1.25	0.69–2.23	0.4588
Varices grade 2–3 (no varices as reference)	**2.87**	**1.74–4.71**	**<0.0001**	**1.84**	**1.02–3.3**	**0.0412**
Unknown, no EGD (no varices as reference)	1.6	0.6–3.54	0.2889	**3.13**	**1.15–7.2**	**0.0129**
Anticoagulation (yes *vs.* no)	**1.81**	**1.18–2.73**	**0.0055**	1.32	0.82–2.09	0.245
Antiplatelets (yes *vs.* no)	0.74	0.44–1.19	0.2384			

Uni- and multivariate Cox regression analyses were used to estimate hazard ratios with 95% confidence intervals for the risk of bleeding. A/B, atezolizumab and bevacizumab; BCLC, Barcelona Clinic Liver Cancer; DT/D, durvalumab with or without tremelimumab; EGD, esophagogastroduodenoscopy; EHS, extrahepatic spread; HR, hazard ratio; MVI, macrovascular invasion. Values in bold denote statistical significance.

Table 4. Frequency and severity of thromboembolic events of A/B *vs.* DT/D (n = 325 *vs.* n = 165).

CTCAE grade	A/B n = 325	DT/D n = 165	*p* value	Odds ratio	95% CI
Any thromboembolism, n (%)	19 (5.8)	10 (6.1)	>0.999	0.96	0.44–2.12
Grade 1–2 (non-severe), n (%)	6 (1.8)	7 (4.2)	0.1403	0.42	0.14–1.28
Grade ≥3 (severe), n (%)	8 (2.5)	3 (1.8)	0.7577	1.36	0.36–5.21
Grade 1, n (%)	0 (0.0)	4 (2.4)			
Grade 2, n (%)	6 (1.8)	3 (1.8)			
Grade 3, n (%)	8 (2.5)	3 (1.8)			
Grade 4, n (%)	0 (0.0)	0 (0.0)			
Grade 5, n (%)	0 (0.0)	0 (0.0)			

Comparisons between groups were performed using the Fisher's exact test. In the A/B group, five patients had a missing CTCAE grade. A/B, atezolizumab and bevacizumab; CTCAE, Common Terminology Criteria for Adverse Events; DT/D, durvalumab with or without tremelimumab.

Thromboembolic events

Thromboembolic events were infrequent and comparably distributed between both treatments (Table 4). A total of 19 thromboembolic events (5.8%) were observed in the A/B group, compared with 10 events (6.1%) in the DT/D group, with no significant difference between the groups (*p* >0.999, OR 0.96, 95% CI 0.44–2.12). Non-severe events (CTCAE grade 1–2) occurred in six (1.8%) *vs.* seven (4.2%) patients, and high-grade events (grade ≥3) in eight (2.5%) *vs.* three (1.8%) patients for A/B and DT/D, respectively (Table 4). For five patients in the A/B group, information on CTCAE grade was missing (Table 4). Time to first thromboembolic event and time to first high-grade thromboembolic event was similar among both groups (Fig. 2).

Reasons for choosing DT/D regimen for systemic treatment

Most patients (92%) were treated with DT/D in the first line. To gain insights into the decision-making process for selecting DT/D, we performed an analysis to capture the reasons for selection (Table 5). We were able to extract the mentioned reasons from the medical records of 112 patients (68%). The most frequently mentioned reason for selecting DT/D was the risk of bleeding (25%), the risk of thromboembolic events (18%), or a combination of bleeding and thromboembolic risk (9%). Further specification indicated that in 43 patients (26%) the bleeding risk was attributed to baseline variceal status. Although wound healing concerns under VEGF-containing therapies were involved in decision-making for 6% of

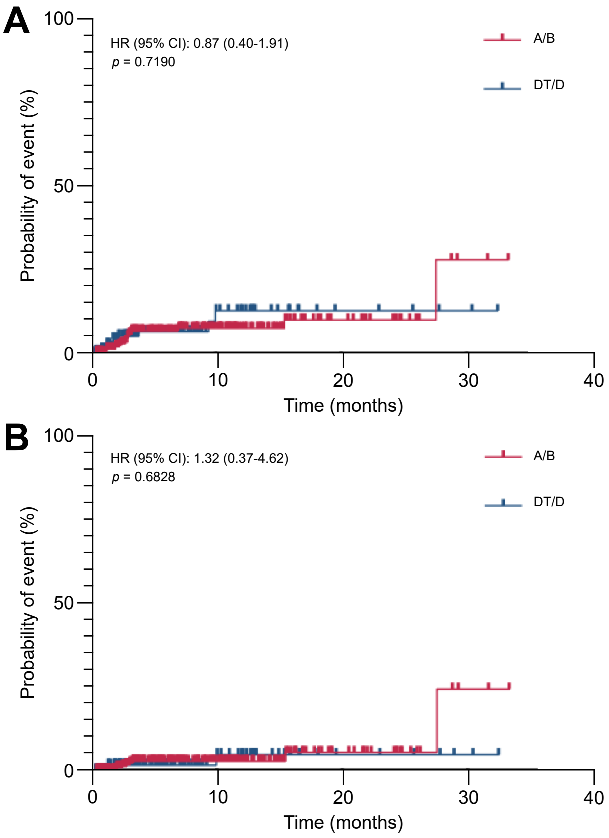

Fig. 2. Kaplan–Meier analysis of time-to-thromboembolism in both cohorts. Time to first thromboembolic episode (A), and time to first high-grade thromboembolic episode are displayed (B). The line represents the cumulative incidence. A/B, atezolizumab/bevacizumab; DT/D, durvalumab + tremelimumab/durvalumab; HR, Hazard Ratio (log-rank).

patients, patient preferences (1%) or simultaneous radiation therapy (3%) were less common reasons. Taken together, concerns about bleeding or thromboembolism under VEGF-containing therapies were the primary reasons for treatment selection in more than half of the patients.

Discussion

Until the approval of DT/D following the positive results of the phase III HIMALAYA trial,[10] only therapeutic regimens targeting VEGF were available for first-line treatment of HCC in Europe, including A/B, Lenvatinib, and sorafenib.[9,30–32] A purely anti-VEGF-free first-line treatment strategy was not available before DT/D.[30] Patients with HCC in clinical practice tend to be sicker than clinical trial populations, where patients with high risk factors for bleeding and thromboembolic events are often excluded. Therefore, the investigation of the vascular side-effect profile in clinical practice is of particular relevance in this at-risk population.

Table 5. Reasons for therapy selection.

Overview of reasons for selecting DT/D	n = 165
**Reasons, n (%)**	
Risk of bleeding	41 (25)
Risk of bleeding and risk of thromboembolic events	15 (9)
Risk of thromboembolic event	30 (18)
Risk of wound healing problem	10 (6)
Risk of thromboembolic event and wound healing problem	2 (1)
Simultaneous radiation	5 (3)
Patient's wish	1 (1)
Other reasons	8 (5)
Unknown	53 (32)

The table illustrates the reasons for choosing therapy with durvalumab, with or without tremelimumab.

In this multicenter European real-world cohort of 490 patients, we observed a higher incidence of bleeding events in patients receiving A/B compared with DT/D (22.8% *vs.* 13.3%, OR 1.92), with severe (grade ≥3) bleeding in 14.5% *vs.* 7.3% of patients. Non-severe events did not differ. Multivariate regression confirmed A/B treatment as an independent predictor of bleeding (HR 2.02, 95% CI 1.21–3.55), alongside prior variceal bleeding and high-grade varices. Time to first bleeding and time to first severe bleeding were both shorter with A/B. Importantly, variceal hemorrhage, the most clinically relevant bleeding complication in patients with cirrhosis, did not differ between groups (A/B: 3.1% *vs.* DT/D: 2.4%). Grade 4 and 5 bleeding events were rare and comparable between groups. Thromboembolic events were similarly infrequent (A/B: 5.8% *vs.* DT/D: 6.1%).

The pattern of some bleeding events suggests an association with anti-VEGF therapy. Mucosal bleeding, for example epistaxis, gingival and vaginal bleeding, as well as hematuria, was more frequent with A/B. These mucosal bleeding events, although more common, are generally manageable and very rarely influence treatment continuation or outcome.

Comparison of bleeding rates between our real-world A/B cohort and the IMbrave150 population reveals findings relevant to clinical practice.[9] Reassuringly, overall bleeding rates were comparable (our study: 22.8% *vs.* 25.2% IMbrave150 bevacizumab-related bleeding), as were variceal hemorrhage (our study: 3.1% *vs.* 2.4% IMbrave150 any grade esophageal varices hemorrhage) and thromboembolic events (our study: 5.8 *vs.* 5.7% IMbrave150 bevacizumab-related thromboembolic events). Severe grade ≥3 bleeding was more frequent in our cohort (14.5% *vs.* 6.4% IMbrave150 bevacizumab-related bleeding grade 3 or 4), probably reflecting patients with higher baseline bleeding risk who have been excluded from the registration trial. Epistaxis, the most common bleeding event in IMbrave150 (10.3%), was less frequent in our cohort (5.2%), likely representing underreporting of minor bleeding episodes. Comparison of our DT/D cohort with the STRIDE arm in HIMALAYA demonstrates consistent overall bleeding rates (13.3% *vs.* 11.3%).[10] Severe grade ≥3 bleeding (7.3% *vs.* 3.9%) and variceal hemorrhage (2.4% *vs.* 0.3%) were more frequent in our cohort, again reflecting a real-world population with more advanced liver disease and portal hypertension. Epistaxis rates were similarly low in both cohorts (1.2% *vs.* 1.5%), consistent with the absence of anti-VEGF activity. Direct comparison of thromboembolic events was not possible, as HIMALAYA did not report these outcomes, to our knowledge.

Our finding of comparable variceal hemorrhage rates between A/B and DT/D aligns with a recent congress abstract from the TriNetX Research Network,[33] which also reported no differences in variceal or gastrointestinal bleeding. However, to our knowledge, overall bleeding rates, severity, and time-to-bleeding have not yet been reported from that cohort.

The higher overall bleeding rate observed with A/B must be interpreted in the context of different treatment periods and baseline characteristics between both cohorts. We selected a

historical cohort of patients treated with A/B, recruited exclusively before the availability of DT/D. This approach was intended to reduce selection bias by directly comparing two concurrent therapies. However, using this method, we cannot rule out bias resulting from comparing cohorts from different time periods. Here, the DT/D group had a higher prevalence of high-grade esophageal varices and more frequently received combined variceal prophylaxis with endoscopic band ligation and non-selective β-blocker therapy. Analysis of medical records revealed that bleeding risk was the most frequently documented rationale for selecting DT/D. Taken together, part of the differences in bleeding events might reflect appropriate real-world selection patterns, where clinicians allocated patients with higher baseline bleeding risks to anti-VEGF-free regimen after approval of DT/D.

Several limitations merit consideration. First, the retrospective design and use of a historical A/B cohort introduce potential selection bias. While this approach was chosen to minimize allocation bias, it cannot be entirely eliminated. Nevertheless, given the superior outcomes observed with A/B compared with sorafenib,[9,13,34] it is likely that only a minority of patients were allocated to alternative therapies. In contrast, when comparing chronologically parallel cohorts of A/B and D/T, a greater degree of selection bias is possible, as patient allocation would occur more directly between the two therapies. A potential additional bias may arise from comparing two therapies across different chronological periods, specifically, an A/B cohort before the approval of DT/D and a DT/D cohort after its approval. Over time, longer survival data for the same therapies have been reported in HCC; for example, lenvatinib reached 13.6 months of mOS in the REFLECT trial,[32] whereas mOS reached 19.0 months in the later LEAP-002 trial.[35] This phenomenon may reflect a more established routine in therapy administration and/or the use of more sequential treatments.[36–39] However, as the primary objective of this study was the evaluation of side effects rather than efficacy, this bias is likely of minor importance, although it cannot be entirely excluded. Thus, while we assume that our analysis is less susceptible to selection bias than chronological direct comparisons, it is not entirely free of it, an inherent limitation of any non-randomized study. The only definitive way to address this issue would be a randomized head-to-head trial, which is not anticipated in the near future. Nevertheless, we believe that the present analysis provides a valuable compromise for generating additional evidence.

Second, the granularity of bleeding characterization was limited compared with prospective trials. Minor bleeding episodes may have been underreported in this study. However, we consider major events to be adequately captured, as patients were treated with close follow-up in specialized tertiary centers. For instance, the IMbrave150 trial reported epistaxis as the most frequently observed bleeding event with 10.3%, an event that typically does not influence treatment decisions in the same way as, for example, intracerebral hemorrhage. A strength of our study is the ability to report variceal bleeding rates, among the most clinically relevant bleeding types. The limited granularity of bleeding data highlights the inherent constraints of the retrospective design, and must be addressed in future prospective studies. As randomized trials are not to be expected on this topic, further analyses with high granularity of baseline characteristics are needed to obtain sufficiently homogeneous data to adequately evaluate the side-effect profiles of both investigated therapies in future meta-analyses. It would be of further interest to clarify whether differences in the time-to-bleeding, or its severity, are attributable to the progression of liver disease or to anti-VEGF effects. We therefore emphasize that prospective studies should use designs capable of distinguishing between these potential causes.

In conclusion, our data suggest that patients receiving A/B experienced more and earlier bleeding events as compared with DT/D. Importantly, variceal bleeding, grade 4 and grade 5 bleeding events, and thromboembolic events did not differ between groups. Although real-world data remain the only available evidence in the absence of prospective randomized trials, they should be interpreted with caution. Most importantly, the study highlights the urgent need for such trials to disentangle selection effects and actual differences in bleeding risk, thereby enabling evidence-based decision-making in HCC therapy.

Affiliations

[1]*Department of Medicine II, University Hospital, LMU Munich, Munich, Germany*[†,‡]*;* [2]*Department of Hepatology and Gastroenterology, Charité-Universitätsmedizin Berlin, Berlin, Germany*[†]*;* [3]*Department of Gastroenterology, Hepatology and Transplant Medicine, University Hospital Essen, University of Duisburg-Essen, Essen, Germany;* [4]*Department of Nuclear Medicine, University Hospital Essen, University of Duisburg-Essen, Essen, Germany;* [5]*Division of Hepatology, Department of Medicine II, University Hospital Würzburg, Würzburg, Germany*[†,‡]*;* [6]*Department of Gastroenterology, Hepatology and Endocrinology, University Hospital Frankfurt, Frankfurt/Main, Germany*[†]*;* [7]*TUM School of Medicine and Health, Department for Internal Medicine II, TUM University Hospital, Technical University of Munich, Munich, Germany* [†,‡]*;* [8]*Department of Internal Medicine I, University Hospital Tübingen, Tübingen, Germany*[†]*;* [9]*M3 Research Center, University Hospital Tübingen, University of Tübingen, Tübingen, Germany*[†]*;* [10]*Department of Medicine I, University Medical Center of the Johannes-Gutenberg University Mainz, Germany*[†]*;* [11]*Division of Gastroenterology, Department of Medicine II, University Hospital Würzburg, Würzburg, Germany*[‡]*;* [12]*Division of Gastroenterology and Hepatology, Department of Medicine III, Medical University of Vienna, Vienna, Austria*[†]*;* [13]*Department of Gastroenterology, Hepatology and Infectious Diseases, Otto-von-Guericke University Hospital Magdeburg, Magdeburg, Germany;* [14]*Gastroenterology, Hepatology, Endocrinology, Rheumatology and Infectious Diseases, Department of Internal Medicine I, University Hospital Regensburg, Regensburg, Germany*[‡]*;* [15]*Department of Gastroenterology, Hepatology and Infectious Diseases, University Hospital Düsseldorf, Düsseldorf, Germany*[†]*;* [16]*Department of Medicine I, University Medical Center, Lübeck, Germany*[†]

Abbreviations

A/B, atezolizumab and bevacizumab; BCLC, Barcelona Clinic Liver Cancer; CCA, cholangiocarcinoma; CTCAE, Common Terminology Criteria for Adverse Events; CTLA-4, cytotoxic T-lymphocyte-associated protein 4; DT/D, durvalumab with or without tremelimumab; EGD, esophagogastroduodenoscopy; EHS, extrahepatic spread; HCC, hepatocellular carcinoma; HR, hazard ratio; ICIs, immune checkpoint inhibitors; mOS, median overall survival; MVI, macrovascular invasion; OR, odds ratio; PD-L1, programmed death-ligand 1; PFS, progression-free survival; TKIs, tyrosine kinase inhibitors; VEGF, vascular-endothelial-growth-factor.

Financial support

This study was initiated by the IMMUreal study group with support by GALC and by the Bavarian Cancer Research Center (BZKF). NBK was supported by the ESMO Research Fellowship, the Bavarian Cancer Research Center, FöFoLe of

LMU Munich funding program (1122), and the German Cancer Consortium. The funding bodies had no role in the design of the study, the collection, analysis, interpretation of data, or the writing of the manuscript.

Conflicts of interest

NBK has received reimbursement of meeting attendance fees and travel expenses from EISAI, lecture honoraria from the Falk Foundation and AstraZeneca and served as advisory board for AstraZeneca, Roche, and Ipsen. He is an unpaid scientific committee member of GASL and UEG and received research support from AstraZeneca, Genentech, and Roche. LSJ has received honoraria for lectures from AstraZeneca, Roche, the Falk Foundation, AbbVie and Boston scientific and travel support from Roche, Biotest and AbbVie. She has served as advisory board member to AstraZeneca, Roche and Boston scientific has received honoraria for lectures from AstraZeneca, the Falk Foundation, IPSEN, Novartis, and Roche and travel support from AstraZeneca, Biotest and Roche. She has served as advisory board or steering committee member to AstraZeneca, Bayer, EISAI, and MSD. KB has received lecture honoraria from Ipsen and reimbursement of conference attendance fees from the Falk Foundation. She is an unpaid scientific committee member of GASL. FrFoe has received honoraria as a speaker and/or consultant from AstraZeneca, Bluejay Therapeutics, BMS, EISAI, Lilly, MSD, Pfizer, Roche, and reimbursement of meeting attendance fees and travel expenses from Merck KGaA and Servier. SJG has received travel support from Ipsen, Gilead, and Merz Therapeutics. MR served as a speaker and/or consultant and/or advisory board member for Astra Zeneca, Bayer, Bristol-Myers Squibb, EISAI, Ipsen, Lilly, MSD, and Roche, and received travel support from Bayer, Bristol-Myers Squibb, Ipsen, and Roche. PB is supported by the Clinician Scientist Fellowship "Else Kröner Research College: 2022 EKFK.05. BS received grant support from AstraZeneca, Eisai, and Ipsen, speaker honoraria from AstraZeneca and Eisai as well as travel support from AbbVie, AstraZeneca, Ipsen, Gilead, and Roche.MV has received payment or honoraria for lectures, presentations, speakers bureaus, manuscript writing, and educational events from AstraZeneca, Incyte, Servier, and MSD. He has received payment for expert testimony from Servier. He has also participated in a Data Safety Monitoring Board or Advisory Board for AstraZeneca, Incyte, Servier, and MSD. AK has received lecture honoraries from Roche Pharma AG, Eisai GmbH, AbbVie Germany AG, Janssen-Cilag GmbH, MSD Sharp & Dohme GmbH, Boston Scientific Corp., Fujifilm Germany, Micro-Tech Germany, and Bayer Pharma AG Germany. CR has received speaker and/or consulting fees and/or travel support from AstraZeneca, AbbVie, Bayer, BMS, Daiichi Sankyo, EISAI, GSK, Incyte, Ipsen, Jazz, Leo Pharma, Lilly, Merck, MSD, Novartis, Pierre Fabre, Roche, Servier, and Taiho. Furthermore, he received institutional research grants by AstraZeneca, Bracco Imaging, and Servier. MP served as a speaker and/or consultant and/or advisory board member for AstraZeneca, Bayer, Bristol-Myers Squibb, Eisai, Ipsen, Lilly, MSD, and Roche and received travel support from Bayer and Bristol-Myers Squibb, Ipsen, and Roche. JUM has received honoraria for lectures, consulting activities, and travel support from the Roche, Eisai, AbbVie, Merz, NovoNordisk, Ipsen, Astra Zeneca, Jansen, and MSD. ENDT reports consultations for AstraZeneca, Bayer, BMS, EISAI, Eli Lilly & Co, MSD, Mallinckrodt, Omega, Pfizer, IPSEN, Terumo, and Roche and employment at Boehringer-Ingelheim and Natera. He reports reimbursement of meeting attendance fees and travel expenses from Arqule, AstraZeneca, BMS, Bayer, Celsion, and Roche, and lecture honoraria from AZ, BMS, and Falk. He has received third-party funding for scientific research from Arqule, AstraZeneca, BMS, Bayer, Eli Lilly, IPSEN, and Roche. AG is advisory board or steering committee member to AbbVie, Advanz, Albireo, Alexion, AstraZeneca, Bayer, BMS, Boehringer, CSL Behring, Eisai, Falk, Gilead, Heel, Intercept, Ipsen, Madrigal, Merz, MSD, Novartis, NovoNordisk, Orphalan, Pfizer, Roche, and Sanofi-Aventis. FPR has received honoraria for lectures, consulting activities and travel support from the Falk Foundation, AbbVie, Gilead, Ipsen, Astra Zeneca, Roche, and Novartis. All other authors declare no conflicts of interest that pertain to this work.

Please refer to the accompanying ICMJE disclosure forms for further details.

Authors' contributions

Designed the study and wrote the manuscript: FPR, NBK. Conducted data analyses: FPR and NBK. Data acquisition, interpretation of results, and preparation of the manuscript: all co-authors. Approved the final version of the manuscript: all authors.

Data availability

Data are available upon request via e-mail to: Reiter_F@ukw.de or najib.benkhaled@med.uni-muenchen.de.

Supplementary data

Supplementary data to this article can be found online at https://doi.org/10.1016/j.jhepr.2026.101818.

References

Author names in bold designate shared co-first authorship

[1] Bray F, Laversanne M, Sung H, et al. Global cancer statistics 2022: GLOBOCAN estimates of incidence and mortality worldwide for 36 cancers in 185 countries. CA Cancer J Clin 2024;74:229–263.

[2] Llovet JM, Kelley RK, Villanueva A, et al. Hepatocellular carcinoma. Nat Rev Dis Primers 2021;7:6.

[3] McGlynn KA, Petrick JL, El-Serag HB. Epidemiology of hepatocellular carcinoma. Hepatology 2021;73(Suppl 1):4–13.

[4] European Association for the Study of the Liver. EASL Clinical Practice Guidelines on the management of hepatocellular carcinoma. J Hepatol 2025;82:315–374.

[5] Singal AG, Llovet JM, Yarchoan M, et al. AASLD Practice Guidance on prevention, diagnosis, and treatment of hepatocellular carcinoma. Hepatology 2023;78:1922–1965.

[6] Qurashi M, Sharma R. Improving hepatocellular carcinoma surveillance in the United Kingdom: challenges and solutions. Lancet Reg Health Eur 2024;43:100963.

[7] **De Toni EN**, **Schlesinger-Raab A**, Fuchs M, et al. Age independent survival benefit for patients with hepatocellular carcinoma (HCC) without metastases at diagnosis: a population-based study. Gut 2020;69:168–176.

[8] Reig M, Forner A, Rimola J, et al. BCLC strategy for prognosis prediction and treatment recommendation Barcelona Clinic Liver Cancer (BCLC) staging system: the 2022 update. J Hepatol 2022;76:681–693.

[9] Finn RS, Qin S, Ikeda M, et al. Atezolizumab plus bevacizumab in unresectable hepatocellular carcinoma. N Engl J Med 2020;382:1894–1905.

[10] **Abou-Alfa GK**, **Lau G**, **Kudo M**, et al. Tremelimumab plus durvalumab in unresectable hepatocellular carcinoma. NEJM Evid 2022;1. EVIDoa2100070.

[11] **Yau T**, **Galle PR**, Decaens T, et al. Nivolumab plus ipilimumab versus lenvatinib or sorafenib as first-line treatment for unresectable hepatocellular carcinoma (CheckMate 9DW): an open-label, randomised, phase 3 trial. Lancet 2025;405:1851–1864.

[12] Ben Khaled N, Mörtl B, Beier D, et al. Changing treatment landscape associated with improved survival in advanced hepatocellular carcinoma: a nationwide, population-based study. Eur J Cancer 2023;192:113248.

[13] Cheng AL, Qin S, Ikeda M, et al. Updated efficacy and safety data from IMbrave150: atezolizumab plus bevacizumab vs. sorafenib for unresectable hepatocellular carcinoma. J Hepatol 2022;76:862–873.

[14] **Rimassa L**, **Chan SL**, Sangro B, et al. Five-year overall survival update from the HIMALAYA study of tremelimumab plus durvalumab in unresectable HCC. J Hepatol 2025;83:899–908.

[15] Sangro B, Chan SL, Kelley RK, et al. Four-year overall survival update from the phase III HIMALAYA study of tremelimumab plus durvalumab in unresectable hepatocellular carcinoma. Ann Oncol 2024;35:448–457.

[16] Alahmari AK, Almalki ZS, Alahmari AK, et al. Thromboembolic events associated with bevacizumab plus chemotherapy for patients with colorectal cancer: a meta-analysis of randomized controlled trials. Am Health Drug Benefits 2016;9:221–232.

[17] Ranpura V, Hapani S, Wu S. Treatment-related mortality with bevacizumab in cancer patients: a meta-analysis. JAMA 2011;305:487–494.

[18] Piseddu I, Jochheim LS, Boettcher K, et al. Early mortality in atezolizumab/bevacizumab for HCC is associated with impaired liver function and alterations of systemic immunity. JHEP Rep 2025;7:101–513.

[19] de Castro T, Jochheim LS, Bathon M, et al. Atezolizumab and bevacizumab in patients with advanced hepatocellular carcinoma with impaired liver function and prior systemic therapy: a real-world experience. Ther Adv Med Oncol 2022;14:17588359221080298.

[20] Sinner F, Pinter M, Scheiner B, et al. Atezolizumab plus bevacizumab in patients with advanced and progressing hepatocellular carcinoma: retrospective multicenter experience. Cancers (Basel) 2022;14:5966.

[21] Sharara AI, Rockey DC. Gastroesophageal variceal hemorrhage. N Engl J Med 2001;345:669–681.

[22] Sogaard KK, Horvath-Puho E, Gronbaek H, et al. Risk of venous thromboembolism in patients with liver disease: a nationwide population-based case-control study. Am J Gastroenterol 2009;104:96–101.

[23] Ben Khaled N, Moller M, Jochheim LS, et al. Atezolizumab/bevacizumab or lenvatinib in hepatocellular carcinoma: multicenter real-world study with focus on bleeding and thromboembolic events. JHEP Rep 2024;6:101065.
[24] **Yamamoto Y**, **Matsui J**, Matsushima T, et al. Lenvatinib, an angiogenesis inhibitor targeting VEGFR/FGFR, shows broad antitumor activity in human tumor xenograft models associated with microvessel density and pericyte coverage. Vasc Cell 2014;6:18.
[25] European Association for the Study of the Liver. EASL clinical practice guidelines: management of hepatocellular carcinoma. J Hepatol 2018;69:182–236.
[26] Cillo U, Vitale A, Grigoletto F, et al. Prospective validation of the Barcelona clinic liver cancer staging system. J Hepatol 2006;44:723–731.
[27] von Elm E, Altman DG, Egger M, et al. The Strengthening the Reporting of Observational Studies in Epidemiology (STROBE) statement: guidelines for reporting observational studies. J Clin Epidemiol 2008;61:344–349.
[28] Castelo-Branco L, Pellat A, Martins-Branco D, et al. ESMO guidance for reporting Oncology real-world evidence (GROW). Ann Oncol 2023;34:1097–1112.
[29] Gross S, Bitzer M, Blodt S, et al. *Z Gastroenterol* Leitlinienreport der S3-Leitlinie Diagnostik und Therapie des Hepatozellularen Karzinoms und biliarer Karzinome - Version 3.0 - Juli 2022, AWMF-Registernummer: 032/053OL, 61; 2023. p. e157–e171.
[30] Reiter FP, Ben Khaled N, Ye L, et al. Advances in pharmacotherapy of hepatocellular carcinoma: a state-of-the-art review. Dig Dis 2022;40:565–580.
[31] Llovet JM, Ricci S, Mazzaferro V, et al. Sorafenib in advanced hepatocellular carcinoma. N Engl J Med 2008;359:378–390.
[32] Kudo M, Finn RS, Qin S, et al. Lenvatinib versus sorafenib in first-line treatment of patients with unresectable hepatocellular carcinoma: a randomised phase 3 non-inferiority trial. Lancet 2018;391:1163–1173.
[33] Bhinder MM, Naeem A, Khudayar M, et al. Comparing the bleeding risk in hepatocellular carcinoma patients treated with atezolizumab/bevacizumab versus durvalumab plus tremelimumab. J Clin Oncol 2025;43(16_suppl):e13882.
[34] Scheiner B, Kang B, Balcar L, et al. Outcome and management of patients with hepatocellular carcinoma who achieved a complete response to immunotherapy-based systemic therapy. Hepatology 2025;81:1714–1727.
[35] Llovet JM, Kudo M, Merle P, et al. Lenvatinib plus pembrolizumab versus lenvatinib plus placebo for advanced hepatocellular carcinoma (LEAP-002): a randomised, double-blind, phase 3 trial. Lancet Oncol 2023;24:1399–1410.
[36] Ben Khaled N, Zarka V, Hobeika B, et al. Therapeutic sequences of systemic therapy after atezolizumab plus bevacizumab for hepatocellular carcinoma: real-world analysis of the IMMUreal cohort. Aliment Pharmacol Ther 2025;61:1755–1766.
[37] Roessler D, Öcal O, Philipp AB, et al. Ipilimumab and nivolumab in advanced hepatocellular carcinoma after failure of prior immune checkpoint inhibitor-based combination therapies: a multicenter retrospective study. J Cancer Res Clin Oncol 2023;149:3065–3073.
[38] **Persano M**, **Rimini M**, Tada T, et al. Sequential therapies after atezolizumab plus bevacizumab or lenvatinib first-line treatments in hepatocellular carcinoma patients. Eur J Cancer 2023;189:112933.
[39] Kobayashi K, Ogasawara S, Takahashi A, et al. Evolution of survival impact of molecular target agents in patients with advanced hepatocellular carcinoma. Liver Cancer 2022;11:48–60.

Keywords: Hepatocellular carcinoma; Immunotherapy; VEGF inhibitors; Bleeding risk; Thromboembolic events.

Received 24 September 2025; received in revised form 27 February 2026; accepted 3 March 2026; Available online 11 March 2026

Journal of Hepatology, Volume *8*

Supplemental information

Multicenter study of bleeding and thromboembolic events with durvalumab ± tremelimumab *vs*. atezolizumab and bevacizumab in advanced HCC

Najib Ben Khaled, Raphael Mohr, Leonie S. Jochheim, Valentina Zarka, Monika Karin, Fabian Artusa, Julia M. Schütte, Vera Himmelsbach, Ursula Ehmer, Katrin Böttcher, Friedrich Foerster, Simon Johannes Gairing, Paula Bark, Alexander Weich, Ignazio Piseddu, Monika Rau, Bernhard Scheiner, Lorenz Balcar, Marino Venerito, Philipp Heumann, Arne Kandulski, Catherine Leyh, Christoph Roderburg, Tom Lüdde, Matthias Pinter, Julia Mayerle, Jens U. Marquardt, Fabian Finkelmeier, Enrico N. De Toni, Andreas Geier, and Florian P. Reiter

Multicenter study of bleeding and thromboembolic events with durvalumab ± tremelimumab *vs.* atezolizumab and bevacizumab in advanced HCC

Najib Ben Khaled, Raphael Mohr, Leonie Jochheim, Valentina Zarka, Monika Karin, Fabian Artusa, Julia M. Schütte, Vera Himmelsbach, Ursula Ehmer, Katrin Böttcher, Friedrich Foerster, Simon Johannes Gairing, Paula Bark, Alexander Weich, Ignazio Piseddu, Monika Rau, Bernhard Scheiner, Lorenz Balcar, Marino Venerito, Philipp Heumann, Arne Kandulski, Catherine Leyh, Christoph Roderburg, Tom Lüdde, Matthias Pinter, Julia Mayerle, Jens U. Marquardt, Fabian Finkelmeier, Enrico N. De Toni, Andreas Geier, Florian P. Reiter

Table of contents

Table S1……………………………………………………………………………………...2

Fig. S1………………………………………………………………………………………...4

Table S1. Bleeding type. Bleeding type of A/B versus DT/D (n=325 vs. n=165). This table annotates the number of patients experiencing a specific bleeding event. For descriptive analyses of bleeding type, all bleeding events were included, with the possibility of patients contributing more than one event (for example a patient experiencing both epistaxis and esophageal variceal bleeding, both events are included).

**Bleeding types of A/B versus DT/D**			
**Bleeding name**	**A/B n=325 patients**	**DT/D n=165 patients**	**Overall n=490 patients**
Epistaxis, n (%)	17 (5.2)	2 (1.2)	19 (3.9)
Esophageal variceal bleeding, n (%)	10 (3.1)	4 (2.4)	14 (2.9)
Lower GI bleeding, n (%)	9 (2.8)	1 (0.6)	10 (2)
GI bleeding unspecified, n (%)	6 (1.8)	3 (1.8)	9 (1.8)
Upper GI bleeding unspecified, n (%)	6 (1.8)	1 (0.6)	7 (1.4)
Bleeding unspecified, n (%)	4 (1.2)	2 (1.2)	6 (1.2)
Gingival bleeding, n (%)	6 (1.8)	0 (0)	6 (1.2)
Gastric ulcer bleeding, n (%)	3 (0.9)	2 (1.2)	5 (1)
Gastric bleeding, n (%)	1 (0.3)	3 (1.8)	4 (0.8)
Upper GI bleeding with reflux esophagitis, n (%)	2 (0.6)	2 (1.2)	4 (0.8)
Duodenal ulcer bleeding, n (%)	3 (0.9)	0 (0)	3 (0.6)
Intracerebral bleeding, n (%)	3 (0.9)	0 (0)	3 (0.6)
Gastric variceal bleeding, n (%)	2 (0.6)	0 (0)	2 (0.4)
Hematuria, n (%)	2 (0.6)	0 (0)	2 (0.4)
Hemorrhoidal bleeding, n (%)	1 (0.3)	1 (0.6)	2 (0.4)
Suspected lower GI bleeding, n (%)	2 (0.6)	0 (0)	2 (0.4)
Bleeding from gastric tumor infiltration, n (%)	1 (0.3)	0 (0)	1 (0.2)

Liver bleeding, n (%)	1 (0.3)	0 (0)	1 (0.2)
Midgut bleeding, n (%)	1 (0.3)	0 (0)	1 (0.2)
Peripheral bleeding after vascular surgery, n (%)	1 (0.3)	0 (0)	1 (0.2)
Rectal varices bleeding, n (%)	0 (0)	1 (0.6)	1 (0.2)
Retroperitoneal hematoma, n (%)	1 (0.3)	0 (0)	1 (0.2)
Suspected upper GI bleeding, n (%)	1 (0.3)	0 (0)	1 (0.2)
Tumor and duodenal bleeding, n (%)	1 (0.3)	0 (0)	1 (0.2)
Tumor bleeding, n (%)	1 (0.3)	0 (0)	1 (0.2)
Vaginal bleeding, n (%)	1 (0.3)	0 (0)	1 (0.2)

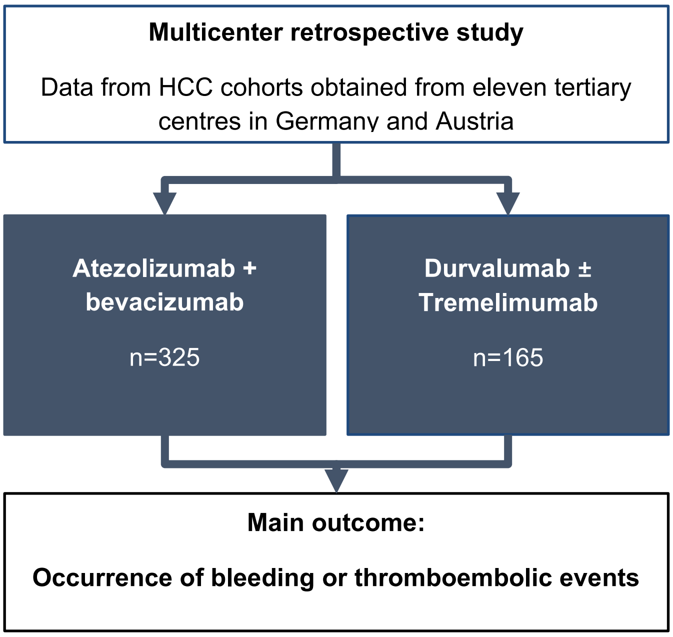

Fig. S1. Study flowchart.
